# Screening of antibiotics and chemical analysis of penicillin residue in fresh milk and traditional dairy products in Oyo state, Nigeria

**DOI:** 10.14202/vetworld.2016.948-954

**Published:** 2016-09-09

**Authors:** Isaac Olufemi Olatoye, Oluwayemisi Folashade Daniel, Sunday Ayobami Ishola

**Affiliations:** 1 Pathogenomic and Proteomic Laboratory, Paul Allen School for Global Animal Health, College of Veterinary Medicine, Washington State University, Pullman, Washington, USA; 2 Department of Veterinary Public Health and Preventive Medicine, University of Ibadan, Ibadan, Nigeria; 3 Analytical Laboratory, Nigerian Institute of Science Laboratory Technology, Ibadan, Nigeria; 4 Department of Chemistry, College of Arts and Science, Washington State University, Pullman, Washington, USA

**Keywords:** milk and milk products, Oyo state, penicillin residues, public health

## Abstract

**Background and Aim:**

There are global public health and economic concerns on chemical residues in food of animal origin. The use of antibiotics in dairy cattle for the treatment of diseases such as mastitis has contributed to the presence of residues in dairy products. Penicillin residues as low as 1 ppb can lead to allergic reactions and shift of resistance patterns in microbial population as well as interfere with the processing of several dairy products. Antibiotic monitoring is an essential quality control measure in safe milk production. This study was aimed at determining antibiotic residue contamination and the level of penicillin in dairy products from Fulani cattle herds in Oyo State.

**Materials and Methods:**

The presence of antibiotic residues in 328 samples of fresh milk, 180 local cheese (wara), and 90 fermented milk (nono) from Southwest, Nigeria were determined using Premi^®^ test kit (R-Biopharm AG, Germany) followed by high-performance liquid chromatography analysis of penicillin-G residue.

**Results:**

Antibiotic residues were obtained in 40.8%, 24.4% and 62.3% fresh milk, wara and nono, respectively. Penicillin-G residue was also detected in 41.1% fresh milk, 40.2% nono and 24.4% wara at mean concentrations of 15.22±0.61, 8.24±0.50 and 7.6±0.60 μg/L with 39.3%, 36.7% and 21.1%, respectively, containing penicillin residue above recommended Codex maximum residue limit (MRL) of 5 μg/L in dairy. There was no significant difference between the mean penicillin residues in all the dairy products in this study.

**Conclusion:**

The results are of food safety concern since the bulk of the samples and substantial quantities of dairy products in Oyo state contained violative levels of antibiotic residues including penicillin residues in concentrations above the MRL. This could be due to indiscriminate and unregulated administration of antibiotics to dairy cattle. Regulatory control of antibiotic use, rapid screening of milk and dairy farmers’ extension education on alternatives to antibiotic prophylaxis, veterinary prescriptions and withdrawal periods are recommended to prevent residues.

## Introduction

The Nigerian dairy industry represents an important component of the agribusiness sector of the economy with great economic, nutritional, and social potentials [1]. Dairy products provide the most important amino acids required for mammalian body growth, development, and repairs [2]. The dairy production is currently gaining commercial improvement in Nigeria. Nigeria is the largest milk producer in West Africa and has the potential of being a major milk producer in Africa with total annual demand estimated at 1.45 billion liters [3,4].

Dairy development in Nigeria is a major component of the Federal Government of Nigeria agricultural transformation agenda. Government and private initiatives are currently making efforts at improving dairy productivity, the processing, packaging, storage, and transportation of milk for internal use and for export. Milk and other dairy products are produced and processed by traditional herdsmen and milk maids. The majority of dairy cattle in Nigeria are reared by the nomadic and semi-nomadic Fulani herdsmen who engage in the indiscriminate use of veterinary drugs for prophylactic and therapeutic purposes in their cattle. Antibiotics, as well as other veterinary drugs, are available over the counter, making drugs easily accessible by livestock farmers as well as the use of chemotherapeutic agents by the nomadic herdsman without veterinary prescription [5]. Tetracyclines β-lactams and aminoglycosides are among the frequently administered antibiotics in livestock production [6]. The indiscriminate use and lack of regulatory monitoring of antibiotics in the traditional dairy production in Nigerian dairy cattle could lead to the presence of residues in dairy products. Antibiotics use in food-producing animals may leave residues in meat, milk, and eggs as a result of failure to observe the withdrawal periods of each drug, extra-label dosages for animals, contamination of animal feed with the excreta of treated animals, or the use of unlicensed antibiotics [7]. Antibiotic residues in foods are of public health concerns due to transfer of antibiotic-resistant bacteria to humans, toxic effects: Carcinogenicity, mutagenicity, nephropathy, hepatotoxicity, bone marrow toxicity, and allergy [8]. Antibiotics in milk also create technical problems in the dairy industry by interfering with the fermentation process through the inhibition of starter cultures used in the production of cheese and yogurt resulting in financial losses [7]. The sale of milk from cows being treated with antibiotics is prohibited in developed countries; milk is routinely tested for the presence of antibiotic residues.

Penicillin is the oldest form of antibiotics used in both man and animals. It is commonly used for the treatment of mastitis and other bacterial infections. Penicillin residue in milk is considered a great public health problem because of consumption of such contaminated milk could result in severe and fatal anaphylactic (allergic) reactions in penicillin-sensitized persons [9,10]. Hence, milk and milk products containing antibiotics beyond safety levels are considered unfit for human consumption [11].

Several studies have reported the presence of antibiotic residues in different foods of animal origin [5,12,13]. However, limited information is available on the occurrence of penicillin residues in bovine milk in Nigeria. More so, dairy development and commercialization efforts in local production require routine residues analysis to protect the consumers from the associated health hazards. This study was aimed at determining the presence of antibiotics and the levels of penicillin residues in milk and milk products from dairy herds in southwest Nigeria.

## Materials and Methods

### Ethical approval

Informed consents of the herdsmen and management of milk collection centers in the study area were obtatined.

### Study location

**Figure 1:**
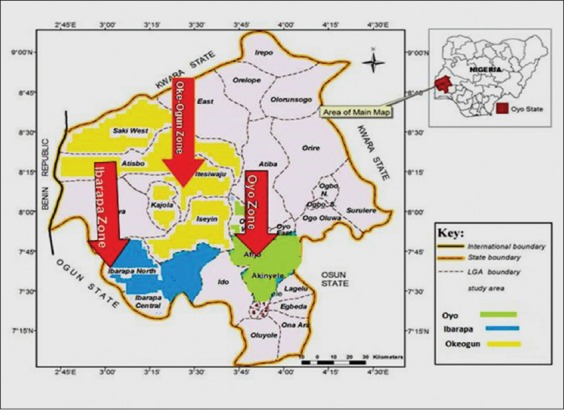
Map of Oyo state showing Ibarapa, Oyo and Oke-Ogun zone.

This study was carried out on bulk tank fresh milk from dairy collection centers and nono and wara from neighborhood milk maids in Ibarapa, Oyo and Oke-Ogun of Oyo state, Nigeria. National Dairy Development Project (public-private-partnership) designates Oyo, Oke-Ogun and Ibarapa zones (with derived savannah vegetation) where the majority of cattle in Oyo state are reared for milk collection from herdsmen and milkmaids. Cattle producers and milk maids are trained and registered under a Dairy Development Programme designed to increase yield of local milk production (Figure-1).

### Sample collection

A total of 598 milk and milk products comprising 328 fresh milk, 180 local cheese (wara), and 90 fermented milk (nono) were randomly collected from Ibarapa, Oyo and Oke-Ogun dairy collection centers over a period of 6-week using sterile sample bottles and bags for antibiotic screening. Immediately after collection samples were transported in iced cool box to the laboratory for analysis. At the time of analysis, the samples were brought up to room temperature. The sample size for high-performance liquid chromatography (HPLC) was determined using the Codex (CAC) table of non-compliance prevalence table of residue [14] after initial screening of all the milk obtained.

### Reagents

The chemical reagents include penicillin G analytical standard (Sigma-Aldrich, Germany), acetonitrile, isopropanol, methanol, petroleum ether, deionized water, HPLC grade water, and dibasic potassium phosphate.

### Screening of antibiotic residues in milk

Premi^®^ test antibiotic residue commercial kit (R-Biopharm AG, Germany) used for screening was performed based on the inhibition of the growth of *Bacillus stearothermophilus*. Samples that were positive by Premi^®^ test screening was extracted and cleaned-up to obtain penicillin residue analytes.

### Chromatographic conditions and calibration

The analysis and quantification of the penicillin residues in the analytes were performed at the Chemical Analysis Laboratory of the Nigeria Institute of Science Laboratory Technology, Ibadan using a HPLC machine (Agilent) equipped with a constant flow pump and a variable wavelength ultraviolet detector set at 204 nm and flow rate of 1.5 ml/min. Elution of penicillin from the analyte was done on a nucleosil C-18 (10 μm, 250 mm × 4.0 mm 1D) column with mobile phase; acetonitrile - 0.01 M and 0.025 M KH_2_PO_4_ at pH 3.0 (20:10:70). Injected volume was 20 μg.

**Figure 2:**
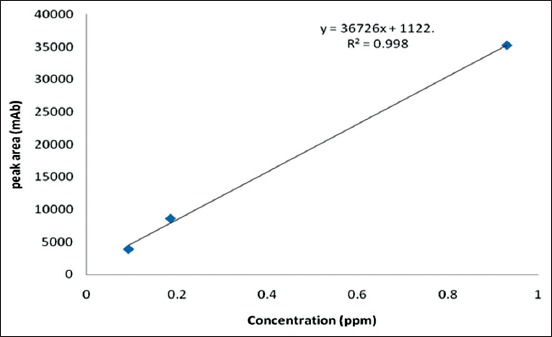
Penicillin analytical standard calibration curve.

**Figure 3:**
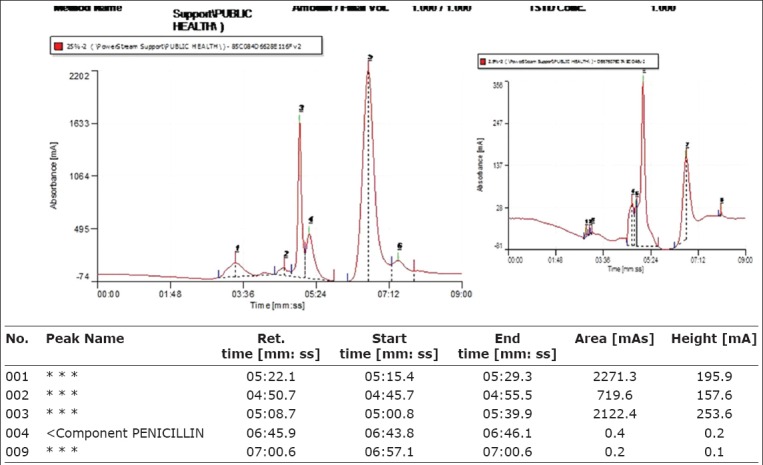
Chromatogram report (peaks) of penicillin standard solution.

**Figure 4:**
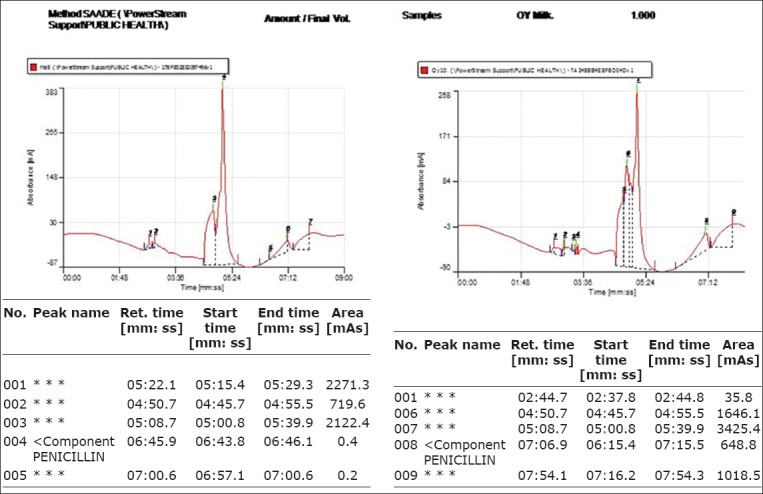
Chromatogram report (peaks) of penicillin residues in milk samples.

Serial dilution of penicillin G analytical standard stock solution (1000 ppm) was carried to obtain 0.4656, 0.1862, and 0.0931 ppm. These concentrations were injected in HPLC machine to obtain corresponding peak areas to obtain calibration curve (Figure-2). The results of the standard concentrations and peak areas were plotted as the standard curve producing a linear regression equation y = 36726x + 1122, where y = Peak area (mAb) and x = Concentration of penicillin (ppm). R^2^ = 0.998. The retention time was 6.36-6.47 min and the detection limit was 0.01 ppm. The chromatographs of penicillin standard and dairy products residue are shown in Figures-3 and 4.

### Sample preparation

Extraction and clean-up procedures described by Shaikh and Moats [15] were employed in this study. The steps involved in the extraction and clean up by homogenizing 2 ml each sample with 2 ml acetonitrile, centrifuged and repeated with 2 ml supernatant and 3 ml acetonitrile w, centrifuged for 5 min at 8000 rpm. Further clean-up of the supernatant was done on Bakerbond-SPE^®^ C_18_ cartridge was conditioned with methanol and water and eluted with 5 ml methanol and 2.5 ml isopropanol. The eluted solution was evaporated to dryness and reconstituted in 1 ml of mobile phase ready for injection HPLC.

### HPLC analysis of samples for penicillin

The mobile phase was filtered through 0.22 μm membrane filter using vacuum pump and regularly degassed by sonication. The HPLC machine was also flushed at interval with blank methanol, and the mobile phase was allowed to run through the machine for equilibration and conditioning during which stable baseline was obtained on the recorder monitor. The column and tubing were regularly checked to ensure leak proof, 20 μl of analyte from each sample (in duplicate) was injected to the column when the machine gave instruction “waiting for pulse injection.” Penicillin was eluted on the C-18 column and resolution occurred in the detector resulting in peaks (chromatographs) shown on the monitor with the peak areas and retention times recorded by the computer recorder. The mean peak areas of the samples corresponding to the retention time between 5 and 6 min obtained from the reference standard were recorded as positive for penicillin. Quantification of residues in the samples was obtained from calculation by substituting the mean peak areas in the linear equation obtained from the calibration curve of the standard.

### Recovery experiment

To validate our procedures, we determined the precision using relative standard deviation (RSD) and percentage recovery. Blank milk samples obtained from untreated cow in the dairy unit of University of Ibadan Teaching and Research were spiked 3 times with 25, 50 and 100 ppm of penicillin-G standard and subjected to extraction, clean-up and HPLC procedures used for the samples.

The mean recovery values for the penicillin obtained from raw milk were between 84.5% and 92.8%, while the RSD ranged between 1.32% and 2.42%. These results showed good linearity and reproducibility with the R^2^ value of 0.998 obtained from the linear curve.

### Statistical analysis

Data were presented in tables, percentages, and graphs with Excel spreadsheet. Mean residue concentrations in different milk products and from different milk collection sites were compared by one-way ANOVA and Student’s t-test (p<0.05) using GraphPad Prism 4 (GraphPad software Inc., San Diego, CA).

## Results

Out of a total of 598, the dairy products screened from the three location 235 (42.2%) were positive for antibiotic residues. The highest prevalence (76.7%) was obtained from Ibarapa (nono) in Oyo state. Results from Oyo zone indicated an overall prevalence of 42.5%. Furthermore, the samples from Oke-ogun yielded the overall prevalence of 37.2%, while samples from Ibarapa had a total prevalence of 49.1% of antibiotic residues. Fresh milk and nono samples yielded more prevalence than wara samples.

The overall mean penicillin residue obtained in milk, nono, and wara was 15.22±0.61, 8.24±0.50, and 7.6±0.60 μg/L, respectively. In Oke-Ogun, zone the mean penicillin concentrations in the different dairy products were 11.58, 6.18 and 8.79 μg/L in fresh milk, wara and nono, respectively, while in oyo zone, the mean penicillin concentrations in the different dairy products were 16.76, 7.32 and 7.91 μg/L in fresh milk, wara and nono, respectively; in Ibarapa zone, the mean penicillin concentrations in the different dairy products were 16.11, 8.91 and 8.11 μg/L in fresh milk, wara and nono, respectively. This result showed that all the dairy products from all the locations contained the residues above maximum residue limits (MRL) (violative levels). The highest mean penicillin residue was in fresh milk samples from Oyo with a mean value of 16.76±0.76 μg/L while the lowest mean penicillin concentration was in wara samples from Oke-ogun with a concentration of 6.18±3.09 μg/L. Mean penicillin residue from Ibarapa was 16.11±1.48, 8.91±0.88, 8.12±0.91 μg/L in fresh milk, wara and nono, respectively. On the overall, 96.3%, 86.1% and 90.3% of fresh milk, wara and nono, respectively, contained penicillin residues above MRL. There were significant differences (p≤0.05) in mean penicillin residue in the different dairy products in each of the three locations.

## Discussion

The increased use of veterinary drugs in dairy herds resulting from the growth in traditional dairy production to meet the increasing population demand for milk and milk products as a major source of protein have become a major global public health concern [8]. There are several national and international regulatory control and monitoring efforts to ensure safety of livestock products meant for human consumption. This study determined the prevalence of antibiotic residues in milk and milk products for public consumption in three selected zone in Oyo state, Nigeria. The results of this study showed a higher prevalence in the samples of antibiotic residues in dairy products. Overall, antibiotic residue prevalence of 42.6% was obtained in the dairy products screened from the three locations in Oyo state. This implies that a large proportion of milk and milk products produced in the study area contained residues of one or more antibiotics. The presence of antibiotics residues could have resulted from self-medication by the herdsmen. This result also indicates that consumers of dairy products in Oyo state are exposed to public health risk associated with consumption of antibiotic residues. However, the prevalence obtained from this study was lower than the 100% of penicillin and amoxicillin residues in goat milk reported by Adetunji and Olaoye [16]. This could mean that goats receive more treatment than dairy cattle herds. In similar studies, other authors [17-19] reported a lower prevalence of 35%, 1.7% and 4% from raw milk, respectively. The different prevalence obtained from fresh milk and milk products could be due to the effect of heat processing of dairy products especially wara as also observed by Zorraquino *et al*. [20]. Furthermore, these could be due to variations in dairy herd management and antibiotic usage in different countries.

The overall mean penicillin residue level in fresh milk 15.22±0.61 μg/L obtained in this study is higher than the international CAC MRL in milk. On screening, 40.8%, 62.3% and 24.4% of the fresh milk, nono and wara samples, respectively, contained antibiotic residues. While Penicillin-G residue was detected in 41.1% fresh milk, 40.2% nono and 24.4% wara at concentrations of 15.22±0.61, 8.24±0.50 and 7.6±0.60 μg/L, respectively, out of which 39.3%, 36.7% and 21.1%, respectively, contained penicillin residue above the recommended Codex MRL of 4.0 μg/L in dairy products.

However, mean penicillin residue concentration obtained in this study is lower than 6240±550 and 59.53±17.91 μg/L reported by Ghidini *et al.*, [21] and Khaskheli *et al.*, [22] in Italy and Pakistan, respectively, in bovine raw milk from. Despite scanty data on penicillin residue in milk from Nigeria, the result of this study shows that penicillin is among the commonly used antibiotics in dairy cattle production in Nigeria. However, about 92.3% of positive milk samples for antibiotic residues contain penicillin residue above Codex MRL. This is higher than the prevalence of 3.1% antibiotics above MRL obtained in raw marketed milk from coastal savannah zone of Ghana [23]. While Khaskheli *et al*. [22] reported 36.5% prevalence of β-lactam antibiotic residues in unprocessed milk in Pakistan.

Hence, milk products from the study area may not meet acceptable food safety standards and could be inimical to consumers’ health. It is known to produce fatal allergic reaction in consumers and contributes to the development and spread of resistant pathogen along the food chain. This could also negatively affect international trade in such products. The findings in this study revealed the importance of antibiotics usage in cattle for dairy production and provided the quantitative analysis of the prevalence and levels of antibiotic residues contamination in fresh milk, nono and wara consumed in three zones from Oyo state, Nigeria. The widespread and unrestricted usage of different antibiotics in food animals without adequate diagnosis, prescription, and supervision by veterinarians contribute greatly to the deposit of residues of these drugs in the dairy product. This was evident by the high prevalence of penicillin residues in these products. The high prevalence of penicillin residues this study could have resulted from the drug being the oldest and commonly used antibiotics in animal production.

## Conclusion

This study showed that substantial quantities of dairy products in Oyo state contain violative levels of antibiotic residues including penicillin. Good management practices and vaccinations of livestock as alternatives to antibiotic prophylaxis should be encouraged. Farmers should be discouraged from administering veterinary drugs without veterinarian prescription. Furthermore, survey covering all the regions in Nigeria should be carried out to fully evaluate the consumer safety of dairy products (fresh milk, nono, and wara) and antibiotic residues in the country. Programs for monitoring the occurrence of residues, consumption of antimicrobial agents and development of resistance in Nigeria are strongly desirable. It is recommended that consumers should drink processed/pasteurized milk products instead of raw milk. There should be proper structuring of dairy production in the country, regular extension education for pastoralist and milk maid, marketers, and processors on good animal husbandry practice to ensure safe milk product supply. Hygienic milk production and processing practices should be promoted among the milk maids and other milk handlers. The improvement of dairy facilities to include laboratory infrastructure for routine testing or monitoring of food-borne microbial and chemical hazards is also recommended.

## Authors’ Contributions

This work was carried out in collaboration between all authors. IOO and OFD designed the study. IOO and SAI reviewed the analytical design and protocols, authors IOO and OFD collected and processed the samples while IOO, OFD and SAI did the analysis. IOO and OFD wrote the first and final drafts of the manuscript. All authors read and approved the final manuscript.
